# Acute Gastric Dilatation After Excessive Alcohol Consumption

**DOI:** 10.7759/cureus.68744

**Published:** 2024-09-05

**Authors:** Koichi Zokumasu, Yuki Natori, Masaki Kawakami

**Affiliations:** 1 Emergency Medicine, Kanto Central Hospital, Tokyo, JPN

**Keywords:** acute gastric dilatation, emergency medicine, frequent vomiting, gastroenterology, heavy alcohol consumption

## Abstract

Acute gastric dilatation (AGD) is a rare but potentially life-threatening condition, commonly associated with mechanical obstructions or systemic conditions such as diabetes and eating disorders. This report describes a 46-year-old man who was brought to the emergency department with frequent vomiting as his chief complaint. He was a habitual heavy drinker and had consumed a large amount of alcohol the night before his admission. He had a previous hospitalization for AGD triggered by excessive alcohol intake a year earlier. Abdominal computed tomography (CT) revealed gastric distension, and his symptoms improved following nasogastric decompression. No significant abnormalities were found during an upper gastrointestinal endoscopy performed after his admission. He was discharged on the fifth day without any complications. This case represents a rare instance of acute gastric dilatation triggered by excessive alcohol consumption. AGD induced by alcohol presents unique clinical challenges compared to typical cases caused by overeating. Specifically, frequent vomiting following heavy alcohol intake can lead clinicians to consider diagnoses such as acute alcohol intoxication or alcoholic ketoacidosis, which may divert attention from the potential for concurrent AGD. This case underscores the importance of considering AGD in the differential diagnosis for patients presenting with frequent vomiting after excessive drinking, illustrating the critical need for careful evaluation to avoid misdiagnosis.

## Introduction

Acute gastric dilatation (AGD) is an uncommon but clinically significant condition characterized by excessive expansion of the stomach. If not promptly recognized and treated, AGD can lead to severe complications, including gastric necrosis, perforation, and respiratory failure due to the distended stomach, sometimes resulting in death [1-3]. There are various reported triggers for AGD. Mechanical obstructions can include gastric cancer or scarring from gastric or duodenal ulcers, and superior mesenteric artery syndrome [4]. Among non-obstructive causes, binge eating has been relatively well-reported [5]. Moreover, systemic conditions like diabetes mellitus and systemic sclerosis have been associated with AGD [6].

To the best of our knowledge, while AGD cases associated with binge eating have been reported, there are no previous reports of AGD attributed to excessive alcohol consumption. We report a case of a 46-year-old man who developed AGD twice within a year, both times following excessive alcohol consumption. Upper gastrointestinal endoscopy during both episodes showed no organic abnormalities, strongly suggesting alcohol as the primary cause.

## Case presentation

A 46-year-old man was brought to the emergency department with the chief complaint of repeated vomiting. He had experienced over 30 episodes of vomiting since the previous night. He did not report any headache, dizziness, chest pain, palpitations, abdominal pain, or diarrhea. Upon arrival, he was alert and oriented. He preferred sitting upright on the stretcher due to nausea, which worsened in the supine position. He had no history of gastric or duodenal ulcers, diabetes mellitus, abdominal surgeries, or binge eating episodes. He had a history of habitual heavy drinking, habitually consuming at least 1.5 liters of alcohol daily, and during social gatherings, he drank to the point of memory loss. He had been admitted with similar symptoms one year prior, and diagnosed with alcohol-induced acute gastric dilatation.

He was 179 cm tall and weighed 68 kg. His body temperature was 37.0°C, blood pressure was 106/74 mmHg, pulse rate was 134 beats per minute, and respiratory rate was 23 per minute. His abdomen was slightly distended but soft, with no tenderness on palpation. Blood tests revealed the following: white blood cell count of 27,500/μL, hemoglobin 16.7 g/dL, platelets 318,000/μL, aspartate aminotransferase (AST) 76 IU/L, alanine aminotransferase (ALT) 35 IU/L, blood urea nitrogen (BUN) 27 mg/dL, creatinine 1.46 mg/dL, sodium 138 mEq/L, potassium 5.5 mEq/L, C-reactive protein 0.36 mg/dL, and D-dimer 0.70 μg/mL. Blood glucose level was 37 mg/dL. The white blood cell count improved to 10,000/μL by the following day of admission. Arterial blood gas analysis showed a pH of 6.97, partial pressure of oxygen (PaO2) of 75.7 mmHg, partial pressure of carbon dioxide (PaCO2) of 20.1 mmHg, bicarbonate (HCO3-) of 4.6 mEq/L, anion gap of 46.2 mEq/L, and lactate was 26 mmol/L (normal range 0.44 to 1.78 mmol/L). Alcoholic ketoacidosis was suggested.

**Figure 1:**
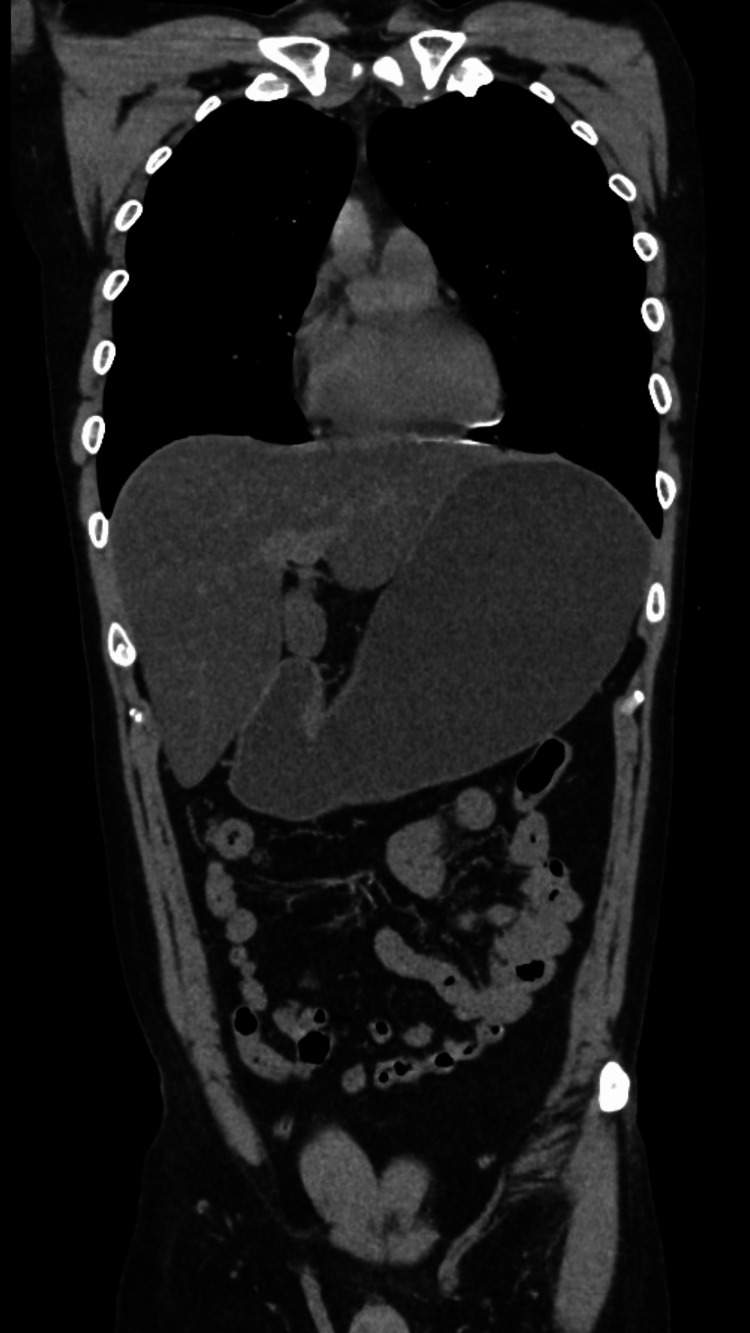
CT scan taken upon emergency admission The patient reported approximately 30 episodes of vomiting prior to the imaging. The scan reveals marked gastric distension with no evident abnormalities distal to the small intestine.

An abdominal CT scan revealed marked gastric distension with no evidence of obstruction in the duodenum or further along the gastrointestinal tract (Figure 1). AGD was strongly suspected.

A nasogastric tube was promptly inserted, and gastric decompression yielded more than 1,500 mL of pure liquid. The patient’s symptoms and signs improved significantly. He was admitted and managed with fasting and intravenous fluid therapy. An upper gastrointestinal endoscopy was performed on the third day of hospitalization, but no organic abnormalities were found that could account for the gastric distension. Nutritional counseling was provided by a registered dietitian during the hospital stay. On the fourth day, oral intake was resumed without recurrence of vomiting. The patient was discharged on the fifth day of hospitalization.

## Discussion

AGD was first documented by Duplay in 1833 [7]. This condition may arise following abdominal surgery, trauma, or from metabolic and psychiatric conditions, such as diabetes and eating disorders, like anorexia and bulimia. Furthermore, there are reported cases where the specific cause of AGD could not be determined, highlighting that the complete pathophysiology of AGD remains elusive [8]. In the present case, the patient had no history of these known predisposing factors, and repeated upper gastrointestinal endoscopies showed no abnormalities, pointing to habitual heavy alcohol consumption as the probable trigger for AGD. While it is commonly understood that the stomach may expand significantly due to binge eating, the intricate mechanisms leading to AGD caused by overeating are still not thoroughly understood. Notably, research focused on identifying risk factors for AGD during nutritional therapy for patients with neurogenic anorexia has failed to pinpoint any specific risks associated with the development of AGD [9].

While most reports of AGD associated with abnormal eating behaviors typically describe excessive solid food intake, cases resulting from significant liquid intake, as seen in this patient, are relatively rare [10,11]. It remains uncertain whether the primary trigger for AGD in this patient was the alcohol itself or the overall volume of liquid consumed. Notably, there are reports of AGD developing in patients with eating disorders where excessive water drinking was a factor, suggesting that the sheer volume of fluid intake, rather than the specific substance consumed, may be critical [12].

Interestingly, both incidents of AGD in this patient occurred in the same month - July, a period when the northern hemisphere, including Japan, experiences a rapid rise in temperatures as it approaches summer. Despite maintaining a consistent pattern of heavy drinking throughout the year, the patient did not develop AGD until the same month a year later. While other gastrointestinal conditions like appendicitis and cholecystitis are more prevalent during warmer periods, suggesting a possible link to environmental factors, whether similar influences contributed to AGD in this case remains entirely speculative [13,14]. This coincidence may simply be random, but it is nonetheless intriguing and warrants a mention, albeit with caution, as a point of interest for future research.

The diagnosis of alcohol-induced AGD is challenging because symptoms like vomiting after heavy alcohol consumption may be initially interpreted as acute alcohol intoxication. This interpretation can lead emergency physicians to conclude their assessment prematurely, potentially foregoing further diagnostic imaging that could reveal AGD. Such oversight can delay essential decompression treatments, escalating the risk of severe complications such as gastric necrosis and perforation.

AGD has been predominantly documented in younger patients, with only a few reports involving elderly individuals [15]. Clinically, patients most often present with symptoms and signs of abdominal pain, vomiting, or distention [6,7,16,17]. Nonetheless, there are instances, such as in the present case, where the condition manifests without significant abdominal pain [12,18]. The absence of pain, especially in younger individuals after heavy drinking, may lead clinicians to misinterpret symptoms as acute alcohol intoxication, potentially overlooking more serious underlying conditions such as AGD.

This case highlights the importance of not only diagnosing acute alcohol intoxication when patients present with vomiting after excessive alcohol consumption but it also emphasizes considering AGD in the differential diagnosis. Diagnosing AGD through abdominal X-rays or CT scans is not difficult, and mild cases can often be managed effectively with nasogastric decompression alone. However, delayed treatment can lead to serious complications, such as gastric wall necrosis and perforation, necessitating surgical intervention or potentially resulting in fatal outcomes.

## Conclusions

We report a case of a middle-aged male with no history of abdominal surgery, diabetes, or eating disorders, such as anorexia or bulimia, who developed AGD twice within a year, presumably triggered by excessive alcohol consumption. While AGD cases linked to eating behavior often involve solid food intake, occurrences due to heavy drinking are rare.

This case highlights a particular challenge in diagnosing AGD induced by alcohol. Clinicians may attribute symptoms solely to acute alcohol intoxication and might overlook the need for abdominal imaging or nasogastric tube placement. Such oversight can delay the diagnosis of AGD, increasing the risk of severe complications like gastric wall necrosis and perforation. This case underscores the importance of considering AGD in the differential diagnosis for patients presenting with extensive vomiting following excessive alcohol consumption and serves as a critical reminder of the need for comprehensive evaluation to prevent potentially fatal outcomes.
